# Validation of the predictive value of combined prealbumin and lymphocyte score for prognosis of stage II/III gastric cancer following curative resection

**DOI:** 10.3389/fonc.2026.1650351

**Published:** 2026-02-12

**Authors:** Wei Peng, Yan Tan, Jun Cheng, Zhengchun Wu, Shuangyan Ou, Jia Luo, Hua Xiao

**Affiliations:** 1 Department of Radiation Oncology, Hunan Cancer Hospital and the Affiliated Cancer Hospital of Xiangya School of Medicine, Central South University, Changsha, China; 2 Gastroenterology and Urology Department II, Hunan Cancer Hospital and The Affiliated Cancer Hospital of Xiangya School of Medicine, Central South University, Changsha, China; 3 Department of Hepatobiliary and Intestinal Surgery, Hunan Cancer Hospital and The Affiliated Cancer Hospital of Xiangya School of Medicine, Central South University, Changsha, China; 4 Department of Gastroduodenal and Pancreatic Surgery, Hunan Cancer Hospital and The Affiliated Cancer Hospital of Xiangya School of Medicine, Central South University, Changsha, China

**Keywords:** gastric cancer, prealbumin, lymphocyte, prognosis, nomogram

## Abstract

**Objective:**

Our previous study found that the combined prealbumin and lymphocyte (Co-PaL) score could accurately classify patients into severe, mild to moderate malnutrition and good nutrition, and might be a predictor for prognosis of patients undergoing gastrectomy for stage II/III gastric cancer (GC). The aim of the present study was to validate these findings.

**Methods:**

The medical records of stage II/III GC patients undergoing curative resection in our hospital from January, 2017 to December, 2023 were retrospectively reviewed. Basing on whether the lymphocyte count was <1.5 ×10^9^/L and/or the prealbumin concentration <180 mg/L, patients were assigned a Co-PaL score of 0, 1 or 2, respectively. A nomogram was established basing on independent predictors for OS identified by univariate and multivariate Cox regression analyses. Concordance index and calibration curves were used to evaluate the nomogram. Clinical utility and predictive accuracy were further assessed by net reclassification index (NRI), integrated discrimination improvement (IDI) and decision curve analysis (DCA).

**Results:**

A total of 890 consecutive patients were recruited. Multivariate regression analyses revealed that Co-PaL score, TNM stage, post-operative complications and adjuvant chemotherapy were independent predictors for OS. A nomogram based on these four variables was established. The C-index value obtained for the model was 0.701 (95%CI: 0.672-0.729). The area under the curve (AUC) values to predict the 1- 3- and 5-year survival probabilities were 0.709 (95%CI: 0.662-0.756), 0.728 (95%CI: 0.692-0.764) and 0.734 (95%CI: 0.695-0.7772), respectively. The calibration curves represented fine consistency between the actual and predicted 1-, 3- and 5-year survival probabilities. Compared with TNM staging system, our model demonstrated strong accuracy, discriminative ability, and clinical utility.

**Conclusions:**

The Co-PaL score was a simple and promising predictor for prognosis of patients undergoing gastrectomy for stage II/III GC. The established nomogram showed superiority over TNM staging system in predicting OS.

## Introduction

Gastric cancer (GC) is one of the most frequently diagnosed malignancies, with both the incidence and mortality ranked fifth worldwide (1). Unfortunately, nearly 70% of GC patients in Western countries and China are diagnosed at the locally advanced stage and even at the advanced stage (2–4). At the present time, curative resection (radical gastrectomy and D2 lymphadenectomy) and peri-operative chemotherapy is the recommended standard management for locally advanced GC (LAGC) (5–7). Although GC managements have dramatically improved, the prognosis of LAGC remains unsatisfactory. Recurrences commonly occur the majority of which appear within 2 years after surgery (8). The pathological tumor-lymph node-metastasis (TNM) classification system is the most frequently utilized predictor for prognosis of patients with GC. But in clinical practice, it is fairly common to encounter patients with the same tumor stage but with significantly different long term outcomes. Therefore, it is necessary to investigate further the underlying mechanism(s) of the disease and other influencing factors (9, 10).

There is increasing evidence that a patient’s immune and nutrition status is associated with not only post-operative complications, but also oncological outcomes of patients with various cancers (2, 9, 11–14). Among these, for example, serum albumin and prealbumin concentrations, neutrophil and lymphocyte counts and C reactive protein (CRP), are the most frequently utilized indicators for immune and nutrition status (15–18). In addition, several combined indexes, including the modified Glasgow Prognostic Score (mGPS) and prognostic nutritional index (PNI), have also been proposed and identified to be independent predictors for the prognosis of several types of malignancies (9, 11, 19). In one of our previous studies that involved 731 stage II or III GC patients who undergoing radical gastrectomy, the optimal cut-off values of prealbumin concentration and lymphocyte count for overall survival (OS) were set as 180 mg/L and 1.5 ×10^9^/L determined by X-tile (18). Further univariate analysis and multivariate Cox regression analyses confirmed that both a prealbumin concentration < 180 mg/L and a lymphocyte count < 1.5 ×10^9^/L were confirmed to be significant predictor for disease free survival (DFS) and OS. Thereafter, a new index, the combined prealbumin and lymphocyte (Co-PaL) score, was established by combining the prealbumin and lymphocyte values. The exact calculation method was as follows: patients with a prealbumin concentration < 180 mg/L and a lymphocyte count < 1.5 ×10^9^/L were given a Co-PaL score of 2, indicating severe malnutrition. Patients with one of these conditions were given a Co-PaL score of 1, indicating mild to moderate malnutrition. Patients with neither of these conditions were given a Co-PaL score of 0, indicating good nutrition. Internal validation (subgroup analyses) found that both OS and DFS were significantly different among patients with a Co-PaL score of 0, 1 and 2, separately. But these conclusions have not been unequivocally validated externally. In this retrospective cohort study involving a large sample size from a tertiary center, a nomogram was established to validate the predictive value of the Co-PaL score in stage II or III GC patients after radical gastrectomy.

## Methods

### Study design

**Figure 1:**
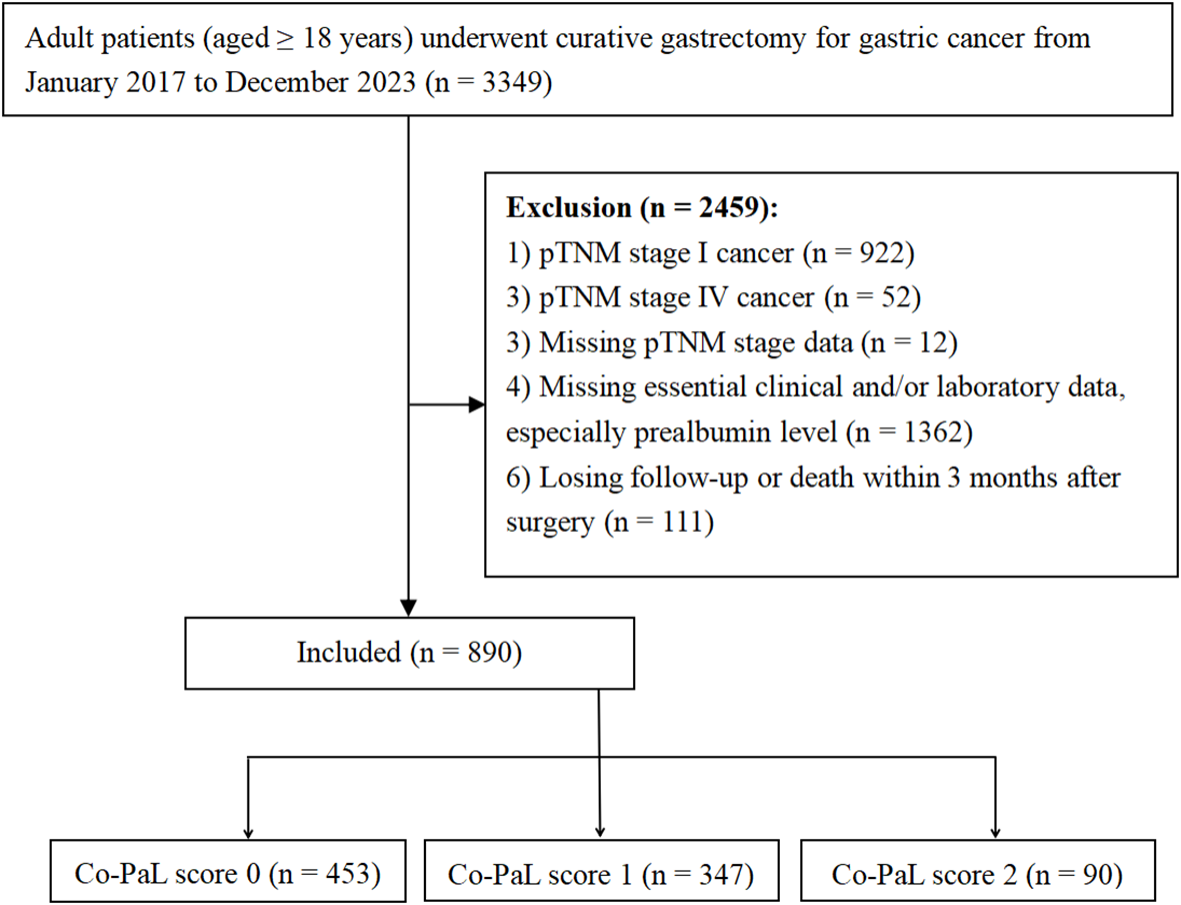
Flow diagram.

The medical data of consecutive adult patients (≥ 18 years old) with pathologically confirmed GC who underwent radical gastrectomy from January, 2017 to December, 2023 in our hospital were retrospectively reviewed. Patients with pTNM stage I or IV disease, missing essential clinical, laboratory and/or pathological data (especially prealbumin level), death or losing follow-up within 3 months after their operations were excluded. A schematic illustration of the study is shown in **Figure 1**, which was approved by the ethics committee of the Hunan Cancer Hospital (No. 72 of quick review in 2025) and complied with the Declaration of Helsinki. Written informed consent to undergo an operation and for the use of their clinic-pathological and follow-up data was obtained from all patients before operation.

### Peri-operative managements and follow-ups

The peri-operative management and follow-up strategies have been documented in our previous studies (11, 18, 20). Briefly, gastric resection and lymph node dissection were performed in accordance with GC treatment guidelines (6, 7). Post-operative morbidity was recorded and classified according to the Clavien-Dindo staging system (21). A small number of patients with T3-4 or N+ diseases received 2-4 cycles of neoadjuvant chemotherapy (NAC), whereas the majority of patients underwent surgery and adjuvant chemotherapy (AC). The most commonly administered drugs for chemotherapy were platinum- and fluorouracil-based regimens, including SOX (S-1 and oxaliplatin) and CapOx (capecitabine and oxaliplatin), et al. (7, 22). Patients were followed up every 3 to 6 months by outpatient examinations or telephone. The cutoff for the follow-up time was December 2024. OS time was measured from the day of a patient’s operation until death or the last follow-up time, whichever occurred first. DFS time was calculated from the operation to GC recurrence, death or the last follow-up time.

### Data collection

The demographic, laboratory, imaging, pathological, operative, adjuvant therapy and follow-up data were retrospectively collected and analyzed. The prealbumin levels and lymphocyte count were collected at the time when the patients’ admission planning to receive surgery. The time intervals were usually 3 to 7 days between laboratory examinations and operation in our hospital. For those undergoing NAC, the basic prealbumin levels and lymphocyte count when their first admission were used to avoid the influence of chemotherapy. The PNI score was generated thus: serum albumin concentration (g/L) + 0.005 × the total lymphocyte count in peripheral blood (/mm3). The neutrophil-to-lymphocyte ratio (NLR) was calculated as the absolute value of neutrophil count divided by the absolute value of lymphocyte. The Co-PaL score was defined according to our previous study (18): a lymphocyte count < 1.5 × 10^9^/L and prealbumin concentration < 180 mg/L was given a score of 1 each, whereas a lymphocyte count ≥ 1.5 × 10^9^/L and prealbumin ≥ 180 mg/L was given a score of 0.

### Statistical analysis

Categorical variables are presented as numbers (%) and compared using a χ^2^ or Fisher’s exact tests, as appropriate. Continuous variables are given as the mean ± standard deviation (SD) and were compared using Student’s *t*-test if normally distributed. The optimal cutoff value for NLR was determined by X-tile (3.6.1 software 20, http://medicine.yale.edu/lab/rimm/research/software.aspx), as previously described (18). Kaplan-Meier curves and the log rank test were utilized to look for differences in survival times. All variables with a *P-*value < 0.05 in the univariate analyses were further entered into a multivariate Cox regression model, to reveal the independent risk factors influencing OS. Data analysis was performed using R (version 4.0.1) or SPSS (version 27.0) software. A difference with a bilateral *P*-value < 0.05 was defined as a statistically significant finding.

### Nomogram establishment and evaluation

A nomogram was constructed utilizing the independent prognosticators for OS in multivariate Cox regression analyses. With respect to each patient, the first line displayed the definite point endowed for each predictor loading on the risk factor axis. Then, the survival probability could be assessed by calculating the sum of each point. Receiver operating characteristic (ROC) curves were plotted and the concordance index (C-index) was generated to evaluate the performance of the nomogram. Calibration curves (1,000 bootstrap resamples) were constructed to assess the predictive ability of the nomogram. Decision curve analysis (DCA) was employed to assess the net benefit of the clinical practicability of the nomogram compared with the TNM stage. Finally, the Net Reclassification Index (NRI) and Integrated Discrimination Improvement (IDI) was used to quantitatively compare the prognostic performance of our model against the commonly used 8^th^ edition TNM staging system.

## Results

### Characteristics of patients

As shown in **Figure 1**, a total of 890 patients were finally recruited and the basic characteristics of the entire cohort are listed in **Table 1**. Taken together, the majority of participants were male (63.5%), with stage III disease (68.8%), undergoing distal sub-total gastrectomy (67.8%), by open procedures (56.0%) and receiving AC (74.6%). The mean age was 57.30 years (23-86), body mass index (BMI) 22.18 kg/m^2^ (range 14.88-36.63), and the mean post-operative hospital stay 11.55 days (range 4-88). One hundred and eighteen patients (13.26%) suffered from grade II or greater post-operative complications defined by the Clavien-Dindo classification system. In total, the Co-PaL score was defined as 0, 1 and 2 in 453 (50.9%), 347 (39.0%) and 90 (10.1%) patients, separately. As shown in **Table 1**, patients with higher Co-PaL scores, were more likely to have worse general conditions (such as an older age, lower BMI, lower hemoglobin, albumin and prealbumin concentrations and lower lymphocyte counts), more likely to have lymph node metastasis and develop complications, but less likely to undergo AC.

**Table 1:** Relationship between Co-PaL scores and clinicopathologic characteristics of patients undergoing radical gastrectomy for stage II/III gastric cancer (n =890).

Variables	Co-PaL score			P-value
	0 (n =453)	1 (n =347)	2 (n =90)	
Sex				0.032
Male	306 (67.55%)	208 (59.94%)	51 (66.67%)	
Female	147 (32.45%)	139 (40.06%)	39 (43.33%)	
Age (years)	55.96 ± 11.23	57.95 ± 11.05	61.61 ± 10.25	<0.001
Body mass index (kg/m^2^)	22.74 ± 3.08	21.73 ± 3.10	21.07 ± 3.69	<0.001
Comorbidities				0.515
Yes	135 (29.80%)	98 (28.24%)	31 (34.44%)	
No	318 (70.20%)	249 (71.76%)	59 (65.56%)	
Peri-operative blood transfusion				<0.001
Yes	58 (12.80%)	86 (24.78%)	35 (38.89%)	
No	395 (87.20%)	261 (75.22%)	55 (61.11%)	
Lymphocyte count (×10^9^/L)	2.04 ± 0.48	1.50 ± 0.67	1.11 ± 0.26	<0.001
Hemoglobin (g/L)	127.10 ± 23.36	114.02 ± 24.69	95.99 ± 24.86	<0.001
Albumin (g/L)	41.84 ± 3.85	39.81 ± 4.35	36.47 ± 4.70	<0.001
Prealbumin (mg/L)	261.15 ± 53.79	216.03 ± 63.33	136.15 ± 34.67	<0.001
PNI score	52.08 ± 4.88	47.33 ± 5.51	42.05 ± 5.06	<0.001
NLR	1.81 ± 0.91	2.51 ± 1.70	4.07 ± 4.30	<0.001
Neo-adjuvant chemotherapy				0.130
Yes	58 (12.80%)	60 (17.29%)	10 (11.11%)	
No	395 (87.20%)	287 (82.71%)	80 (88.88%)	
Operation method				0.998
Open	254 (56.07%)	153 (44.09%)	39 (43.33%)	
Laparoscopy	199 (43.93%)	194 (55.91%)	51 (56.67%)	
Type of resection				0.730
Distal subtotal gastrectomy	306 (67.55%)	236 (68.01%)	61 (67.78%)	
Proximal subtotal gastrectomy	3 (0.66%)	4 (1.15%)	2 (2.22%)	
Total gastrectomy	144 (31.79%)	107 (30.84%)	27 (30.00%)	
Depth of invasion*				0.709
T1	16 (3.53%)	12 (3.46%)	1 (1.11%)	
T2	55 (12.14%)	32 (9.22%)	11 (12.22%)	
T3	116 (25.61%)	89 (25.65%)	26 (28.89%)	
T4	266 (58.72%)	214 (61.67%)	52 (57.78%)	
Lymph node metastasis stage*				0.035
N0	77 (17.00%)	70 (20.17%)	9 (10.00%)	
N1	96 (21.19%)	59 (17.00%)	17 (18.89%)	
N2	126 (27.81%)	83 (23.92%)	19 (21.11%)	
N3	154 (34.00%)	135 (38.90%)	45 (50.00%)	
TNM stage*				0.144
II	148 (32.67%)	110 (31.70%)	20 (22.22%)	
III	305 (67.33%)	237 (68.30%)	70 (77.78%)	
Post-operative complications †				0.039
Yes	48 (10.60%)	53 (15.27%)	17 (18.89%)	
No	405 (89.40%)	294 (84.73%)	73 (81.11%)	
Peri-operative blood transfusion				
Yes				
No				
Post-operative hospital stay (days)	11.48 ± 6.56	11.80 ± 5.87	10.89 ± 3.75	0.426
Adjuvant chemotherapy				<0.001
Yes	363 (80.13%)	243 (70.03%)	58 (64.44%)	
No	90 (19.87%)	104 (29.97%)	32 (35.56%)	
Peri-operative chemotherapy cycles				0.002
0	82 (18.10%)	92 (26.51%)	32 (35.56%)	
1-5	167 (36.87%)	107 (30.84%)	24 (26.67%)	
≥ 6	204 (45.03%)	148 (42.65%)	34 (37.78%)	

Data are presented as mean ± standard deviation or number (%).

Co-PaL, the combined prealbumin and lymphocyte; NLR, neutrophil-to-lymphocyte ratio; PNI, prognostic nutritional index.

*Tumor stages are based on 8th edition of the Union for International Cancer Control TNM classification.

†Defined as Clavien-Dindo grade II or greater.

### Risk factors for prognosis

A total of 387 cases of deaths (43.5%) were recorded within the median follow-up time of 38 months (range, 4-95), with an estimated median OS (mOS) of 76 months for the entire cohort of 890 patients.

As shown in **Table 2**, univariate analysis revealed that age, BMI, the Co-PaL and PNI scores, TNM stage, blood transfusion, complications and AC were potential risk factors for OS (all *P* < 0.05). Further multivariate Cox regression analysis containing these variables confirmed that only the Co-PaL score, TNM stage, complications and AC were significant predictors. In contrast, age, BMI, the PNI score and blood transfusion all lost their significance after multivariate analysis (all *P* > 0.05). The estimated mOS in patients with Co-PaL scores of 0, 1 and 2 were not available (NA), 63 and 45 months, respectively (*P* < 0.001). Compared to patients with a Co-PaL score of 0, those with a score of 1 (hazard ratio (HR): 1.293, 95% confidence interval (CI): 1.042-1.604, *P* = 0.020) and 2 (HR: 1.519, 95% CI: 1.108-2.083, *P* = 0.009) had significant poorer prognosis.

**Table 2:** Univariate and multivariate analyses of prognostic factors for overall survival after radical resection of stage II/III gastric cancer (n =890).

Variables	N (%)	Median OS (months)	UV *P* value	MV HR (95% CI)	MV *P* value
Gender			0.848		
Male	565 (63.48%)	87.0			
Female	325 (36.52%)	66.0			
Age (years)			0.008		0.484
≥ 65	248 (27.87%)	51.0		Reference	
< 65	642 (72.13%)	NA		1.062 (0.843-1.337)	
Body mass index (kg/m^2^)			0.006		0.055
≥ 25.0	156 (17.53%)	NA		Reference	
18.5-24.9	626 (70.34%)	72.0		1.354 (1.004-1.825)	
< 18.5	108 (12.13%)	48.0		1.559 (1.058-2.299)	
Comorbidities			0.875		
Yes	264 (29.66%)	87.0			
No	626 (70.34%)	74.0			
Pre-treatment hemoglobin (g/L)			0.069		
≥ 100	673 (75.62%)	90.0			
< 100	217 (24.38%)	54.0			
Co-PaL score			<0.001		0.011
0	453 (50.90%)	NA		Reference	
1	347 (38.99%)	63.0		1.293 (1.042-1.604)	
2	90 (10.11%)	45.0		1.519 (1.108-2.083)	
PNI score			0.002		0.665
≥ 50	416 (46.74%)	NA		Reference	
40-50	416 (46.74%)	57.0		1.226 (0.973-1.546)	
< 40	58 (6.52%)	51.0		1.397 (0.973-2.006)	
NLR			0.053		
< 3.25	750 (84.27%)	84.0			
≥ 3.25	140 (15.73%)	51.0			
pTNM stage *			<0.001		<0.001
II	278 (31.24%)	NA		Reference	
III	612 (68.76%)	42.0		3.500 (2.651-4.620)	
Peri-operative blood transfusion			0.006		0.955
No	711 (79.89%)	90.0		Reference	
Yes	179 (20.11%)	46.0		0.967 (0.748-1.250)	
Post-operative complications †			0.001		0.007
No	772 (86.74%)	87.0		Reference	
Yes	118 (13.26%)	38.0		1.455 (1.106-1.914)	
Adjuvant chemotherapy			<0.001		<0.001
No	226 (25.39%)	38.0		Reference	
Yes	664 (74.61%)	NA		0.561 (0.451-0.699)	

Data are presented as mean ± standard deviation or number (%).

CI, confidence interval; Co-PaL, the combined prealbumin and lymphocyte; HR, hazard ratio; MV, multivariate analysis; NA, not available; NLR, neutrophil-to-lymphocyte ratio; OS, overall survival; PNI, prognostic nutritional index; UV, univariate analysis.

*Tumor stages are based on 8th edition of the Union for International Cancer Control TNM classification.

†Defined as Clavien-Dindo grade II or greater.

**Figure 2:**
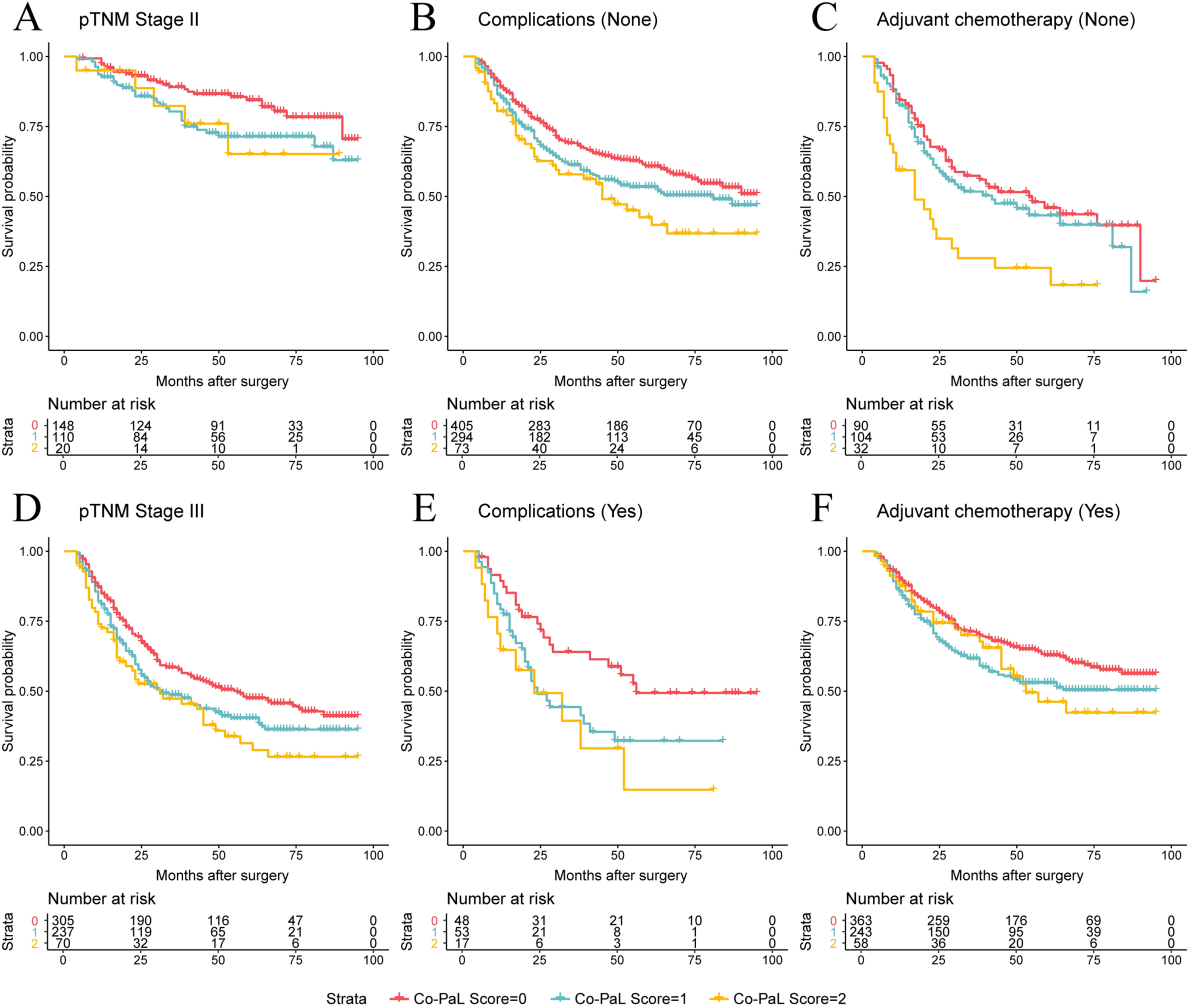
Over survival curves in 890 patients who underwent curative resection for stage II/III gastric cancer stratified by Co-PaL score in TNM stage II or III (**(A, D)**, *P* = 0.085 and 0.008), with or without post-operative complications (**(B, E)**, *P* = 0.021 and 0.008), receiving adjuvant chemotherapy or not (**(C, F)**, *P* = 0.003 and 0.021), respectively.

A tendency of poorer prognosis was found for patients with higher Co-PaL scores in stage II disease by subgroup analysis, but the apparent difference did not reach statistical significance (**Figure 2A**, P = 0.085). Whereas the Co-PaL score could clearly classify patients into low, medium and high risk groups in pTNM stage III disease (**Figure 2D**, P = 0.008), in patients developing post-operative complications or not (**Figures 2B, E**, *P* = 0.021 and 0.008, respective) and receiving AC or not (**Figures 2C, F**, *P* = 0.003 and 0.021, respective). As stratified by peri-operative chemotherapy cycles, patients with Co-PaL score of 2 had significantly worse prognosis comparing to those with score of 0 or 1 and did not receive any cycles of AC (*P* = 0.002). The differences were not significant in those underwent 1-5 or ≥ 6 cycles of peri-operative chemotherapy (*P* = 0.128 and 0.069, separately) (**Supplementary Figure 1**).

The Co-PaL score was also identified as a significant risk factor for DFS (**Supplementary Table 1**). Compared to patients with a Co-PaL score of 0, those with a score of 1 (HR: 1.268, 95% CI: 1.023-1.571, *P* = 0.030) and 2 (HR: 1.537, 95% CI: 1.123-2.102, *P* = 0.007) had significantly poorer DFS. The PNI score lost its significance for DFS after multivariate analysis again (*P* = 0.589).

### Nomogram establishment and evaluation

**Figure 3:**
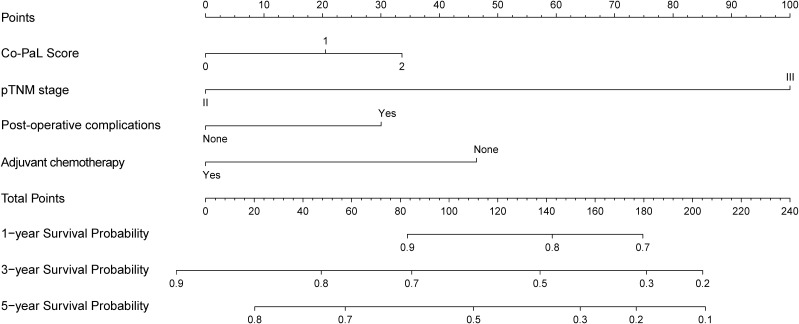
A nomogram to predict 1-, 3- and 5-year overall survival probability of stage II/III gastric cancer patients undergoing curative resection.

**Figure 4:**
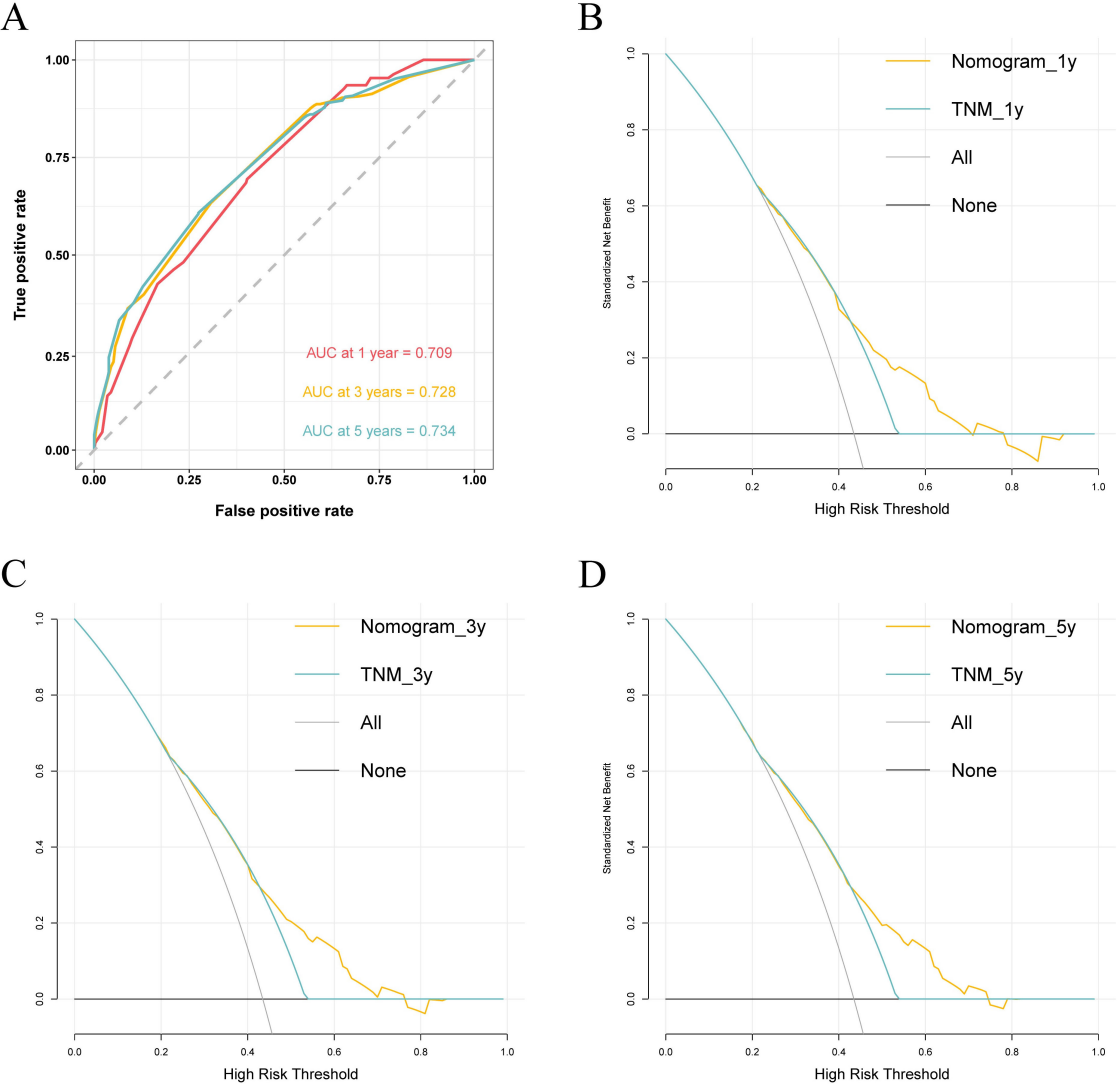
Receiver operating characteristic (ROC) area under the curve (AUC) values of the nomogram for predicting 1-, 3- and 5-year survival probability of stage II/III gastric cancer patients undergoing curative resection **(A)**. Decision curve analysis (DCA) of the nomogram were plotted basing on the 1-, 3- and 5-year overall survival, respectively (**B-D**).

**Figure 5:**
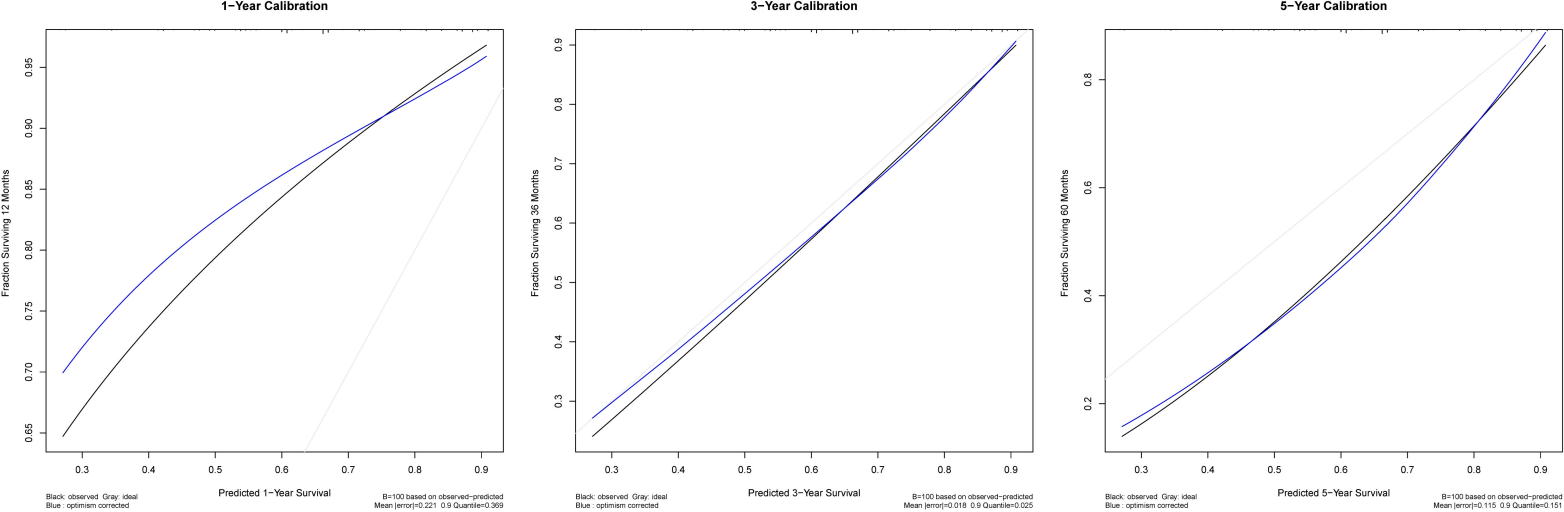
Calibration curves for the nomogram to predict 1-, 3- and 5-year overall survival for stage II/III gastric cancer patients undergoing radical gastrectomy.

In accordance with the multivariate analyses, the Co-PaL score, TNM stage, complications and AC were used to establish a nomogram to assess the OS probability (**Figure 3**). As shown in **Figure 4A**, the area under the curve (AUC) values to predict the 1- 3- and 5-year survival probabilities were 0.709 (95% CI: 0.662-0.756), 0.728 (95% CI: 0.692-0.764), and 0.734 (95% CI: 0.695-0.7772), respectively. The corresponding Harrell C-index value of the model was 0.701 (95% CI: 0.672-0.729). Taken together, the nomogram showed good predictive ability. As shown in **Figure 5**, calibration curves displayed fine consistency between the actual and predicted survival probabilities at 1-, 3- and 5-years. The DCA curves showed the established predictive model was superior in predicting OS rates at 1-, 3- and 5-years compared to the commonly used TNM tumor classification system (**Figures 4B–D**). In addition, the NRI value was 0.294 (95% CI: 0.165-0.423) and the IDI value was 0.030 (95%CI: 0.019-0.042), with both *P*-values < 0.01 comparing with the 8^th^ TNM staging system. As a result, the newly established nomogram had significantly improved the predictive accuracy.

## Discussion

In our previous study (18), both a lymphocyte count < 1.5 ×10^9^/L and a prealbumin concentration < 180 mg/L were identified as independent unfavorable predictors for OS of stage II/III GC patients following curative resection. Thereafter, the newly established Co-PaL score, could accurately classify patients into poor, mild to moderate, and good nutritional statuses and was a potential predictor for prognosis. In order to validate these findings, we conducted this retrospective study by analyzing the medical data of 890 consecutive patients with stage II or III GC. Except for the well-known tumor stage, post-operative complication and AC, the Co-PaL score was also identified as a significant risk factor for OS. Subgroup analyses found that regardless of the tumor stage, complications and AC, the Co-PaL score was significantly associated with prognosis. Furthermore, the established nomogram based on the above mentioned 4 predictors showed a good ability to predict the oncological outcomes. The calibration curves indicated satisfactory consistency between the actual and predicted survival probabilities at 1-, 3- and 5-years. Finally, DCA of the nomogram showed superiority compared to the TNM classification system. As listed in **Supplementary Table 2**, the basic characteristics between the development and validation cohort were significantly different, including age, BMI, prealbumin and albumin concentrations, the tumor stage and complications. Validation through a new dataset with a significant difference of basic characteristics ensured the promising predictive value of the Co-PaL score for prognosis in stage II/III GC patients who underwent gastrectomy.

The TNM stage was the most well-established predictor for treatment strategy and prognosis of GC (5–7, 23). As shown in **Figure 2**, the established nomogram revealed that stage III disease was 100 points higher compared to stage II disease, which was obviously higher than the other 3 variables. Thus, the pTNM stage was identified as the most significant predictor for prognosis in the present study. Post-operative complications, especially infections, are also well-known adverse indicators for the prognosis of various cancer types (2, 24–27). Possible explanations include that post-operative complications might delay AC, adversely impact the compliance of AC, cause systemic inflammation and weaken the hosts’ immunity against cancer cells (2, 24–28). To reduce and/or delay recurrence after resection, AC has been recommended as the standard management for LAGC worldwide (5–7). Consistent with previous study findings, AC has been confirmed as a protective factor for long-term survival in the present study (22, 27). But in clinical practice, it is not uncommon to come across patients who refuse to undergo AC, or could not finish the planned AC regimens for various reasons, such as delayed recovery from surgery, frailty and AC induced toxicity, and so on. Even in the prospective phase 3 CRITICS study, about a quarter of patients did not receive any chemotherapy or chemo-radiotherapy postoperatively as planned. In addition, another 25% of patients did not complete the planned chemotherapy regimen (29).

Several studies have found that the completeness of AC affected the prognosis of LAGC (30, 31). In the well-known CLASSIC study (30), *post hoc* analysis found that patients receiving 6 or more cycles of AC had a significantly better prognosis than those receiving less than 6 cycles. Our previous studies found that poorer nutritional statuses, which were assessed by PNI or BMI, independently affected the completeness of AC (31, 32). In the present study, patients with higher Co-PaL scores, were found to be less likely to receive AC (80.13%, 70.03% and 64.44% in those with Co-PaL score of 0, 1 and 2, respectively, *P* < 0.001). In addition, 45.03%, 42.65% and 37.78% of patients with Co-PaL scores of 0, 1 or 2 completed at least 6 cycles of peri-operative chemotherapy (*P* = 0.002). Thus, the Co-PaL score, which indicate nutritional and immune statuses, might serve as an indicator for compliance of AC, a suggestion which requires further research. Another previous study proved that oral nutritional supplements could improve nutritional outcomes and the completion of planned therapy (33). As a result, it is reasonable to suppose that such strategies might improve the long term outcomes of LAGC, but further prospective studies are required to verify this conjecture.

Malnutrition occurs commonly in cancer patients, especially in those with digestive system cancers, such as GC (34). The unique anatomical structure of GC results in an imbalance of nutrient intake and bodily requirements which might partly explain the underlying reasons. Malnutrition, in turn, adversely impacts immune functions. For example, malnutrition may result in an immune-suppressive micro-environment and increase the rate of invasive tumor formation (35). Nowadays, more and more evidence has accumulated showing that malnutrition and the impaired immune status is not only related to increased post-operative complications and prolonged duration of hospital stays, but also to increased chemotherapy-induced toxicity, influenced the compliance of chemotherapy, and as a result led to a poorer prognosis (9, 11, 14, 15, 31). Albumin, prealbumin, lymphocyte and CRP are the most commonly utilized indicators for immunological and nutritional statuses (15–18). But the conclusions of researchers have not always been uniquely consistent. In a retrospective study of 4,732 GC patients, a lower prealbumin concentration (< 15 mg/dL and 15-22 mg/dL vs. ≥ 22 mg/dL) was identified as significant risk factors for OS. Further analysis found that the prealbumin concentration was associated with other cause survival, instead of cancer specific survival (16). In one of our previous studies involving 731 stage II/III GC patients who underwent operations, a lower prealbumin concentration (< 180 mg/L), instead of albumin, was confirmed as an independent poor predictor for both OS and DFS (18). The possible explanation was that prealbumin has a significantly smaller body pool and shorter half-life than albumin. Thus prealbumin might serve as a more sensitive indicator for nutritional status.

On the other hand, the lymphocyte count is a simple and widely used indicator to assess immunity. Decreased lymphocyte numbers generally represents an immunosuppressed condition to the attack and elimination of cancer cells, resulting in an unsatisfactory prognosis of various cancer types (36, 37). In one of our previous research projects, 390 GC patients were enrolled from 3 tertiary hospitals who underwent radical gastrectomy and whose peripheral lymphocyte subsets were examined. A T lymphocyte count < 0.84 × 10^9^/L was confirmed to be a significant adverse predictor both in the development and validation groups. The ability of antigen directed cyto-toxicity of the T lymphocyte, which plays an essential role in cellular adaptive immunity fighting cancers, might partly explain our findings (38). But further prospective studies are still awaited to confirm these findings and explore the underlying mechanisms.

There were a number of limitations to the present study. First, it was retrospective in nature and conducted in a single-center; thus, selective bias was likely inevitable. Second, only about 10% of patients (90/890) were assigned a Co-PaL score of 2, which represented severe malnutrition in the present study. The relatively small numbers of patients in some subgroups might have had an impact on the robustness of our conclusions. Third, although the majority of relapses of LAGC occurred within 36 months following resection (39), the relatively short follow-up duration of 38 months may have been inadequate to gather and analyze late relapses and deaths. Lastly, but by no means least, external validation with large sample size from geographically/institutionally distinct centers is still required to verify our findings, especially in those patients from Western countries, given that management strategies for LAGC differ significantly among the Western and Eastern countries.

In conclusion, this study confirmed that the lymphocyte and prealbumin based Co-PaL score was a promising prognosticator in stage II or III GC after radical gastrectomy. The established nomogram incorporating the Co-PaL score exhibited a satisfactory predictive ability to predict the survival probability at 1-, 3- and 5-years. Further external validation is still needed to assess the accuracy of this nomogram.

## Data Availability

The raw data supporting the conclusions of this article will be made available by the authors, without undue reservation.

## Supplementary material

Supplementary Figure 1 Over survival curves in 890 patients who underwent curative resection for stage II/III gastric cancer stratified by Co-PaL score in patients receiving peri-operative chemotherapy cycle ≥ 6 **(A)** 1-5 **(B)** or 0 **(C)**.
