# Supplementary figures and images for: Validation of the predictive value of combined prealbumin and lymphocyte score for prognosis of stage II/III gastric cancer following curative resection
Source: Front Oncol. 2026 Feb 12;16:1650351. doi: 10.3389/fonc.2026.1650351 (PMC12935669; doi:10.3389/fonc.2026.1650351)

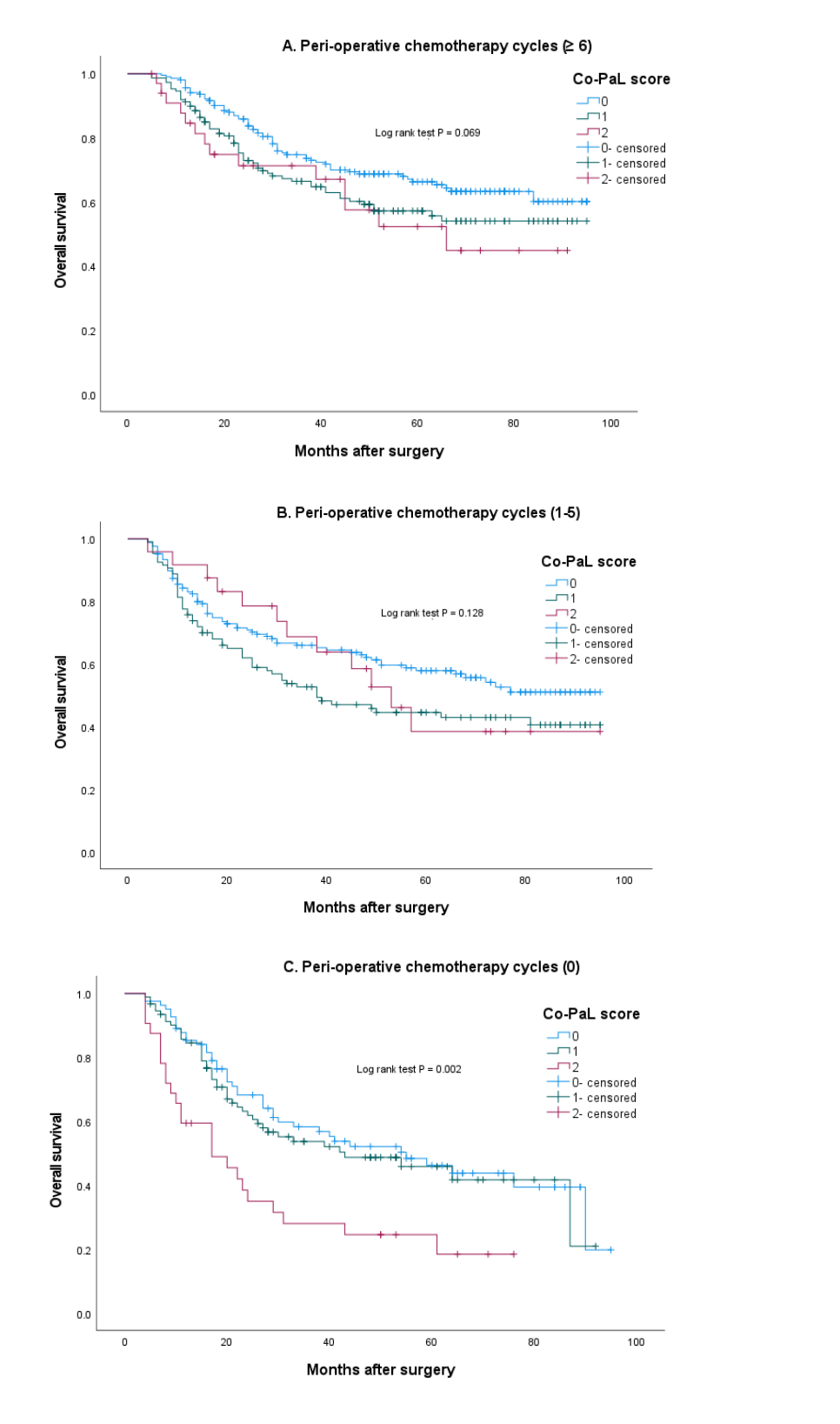

Supplement: Supplementary Figure 1 — Over survival curves in 890 patients who underwent curative resection for stage II/III gastric cancer stratified by Co-PaL score in patients receiving peri-operative chemotherapy cycle ≥ 6 (A) 1-5 (B) or 0 (C). [file Image1.tif]
