# Supplementary material for: Validation of the predictive value of combined prealbumin and lymphocyte score for prognosis of stage II/III gastric cancer following curative resection
Source: Front Oncol. 2026 Feb 12;16:1650351. doi: 10.3389/fonc.2026.1650351 (PMC12935669; doi:10.3389/fonc.2026.1650351)
Supplement: Supplementary file 2 [file Table1.doc]

| **Supplementary Table 1.** Univariate and multivariate analyses of prognostic factors for disease free survival after radical resection of stage II/III gastric cancer (n =890) | | | | | |
| --- | --- | --- | --- | --- | --- |
| Variables | N (%) | Median DFS (months) | UV  *P* value | MV  HR (95% CI) | MV  *P* value |
| Gender |  |  | 0.470 |  |  |
| Male | 565 (63.48%) | NA |  |  |  |
| Female | 325 (36.52%) | 60.0 |  |  |  |
| Age (years) |  |  | 0.568 |  |  |
| ≥ 65 | 248 (27.87%) | 74.0 |  |  |  |
| < 65 | 642 (72.13%) | NA |  |  |  |
| Body mass index (kg/m^2^) |  |  | 0.015 |  | 0.088 |
| ≥ 25.0 | 156 (17.53%) | NA |  | Reference |  |
| 18.5-24.9 | 626 (70.34%) | 77.0 |  | 1.313 (0.981-1.757) |  |
| < 18.5 | 108 (12.13%) | 42.0 |  | 1.486 (1.015-2.174) |  |
| Comorbidities |  |  | 0.341 |  |  |
| Yes | 264 (29.66%) | NA |  |  |  |
| No | 626 (70.34%) | 70.0 |  |  |  |
| Pre-treatment hemoglobin (g/L) |  |  | 0.016 |  | 0.907 |
| ≥ 100 | 673 (75.62%) | NA |  | Reference |  |
| < 100 | 217 (24.38%) | 37.0 |  | 0.978 (0.730-1.309) |  |
| Co-PaL score |  |  | <0.001 |  | 0.012 |
| 0 | 453 (50.90%) | NA |  | Reference |  |
| 1 | 347 (38.99%) | 57.0 |  | 1.268 (1.023-1.571) |  |
| 2 | 90 (10.11%) | 28.0 |  | 1.537 (1.123-2.102) |  |
| PNI score |  |  | 0.005 |  | 0.589 |
| ≥ 50 | 416 (46.74%) | NA |  | Reference |  |
| 40-50 | 416 (46.74%) | 50.0 |  | 1.104 (0.881-1.384) |  |
| < 40 | 58 (6.52%) | 38.0 |  | 1.397 (0.655-1.576) |  |
| NLR |  |  | 0.058 |  |  |
| < 3.25 | 750 (84.27%) | 83.0 |  |  |  |
| ≥ 3.25 | 140 (15.73%) | 39.0 |  |  |  |
| pTNM stage * |  |  | <0.001 |  | <0.001 |
| II | 278 (31.24%) | NA |  | Reference |  |
| III | 612 (68.76%) | 33.0 |  | 3.622  (2.735-4.798) |  |
| Peri-operative blood transfusion |  |  | 0.006 |  | 0.721 |
| No | 711 (79.89%) | NA |  | Reference |  |
| Yes | 179 (20.11%) | 30.0 |  | 1.033 (0.758-1.408) |  |
| Post-operative complications † |  |  | 0.010 |  | 0.015 |
| No | 772 (86.74%) | NA |  | Reference |  |
| Yes | 118 (13.26%) | 30.0 |  | 1.417 (1.070-1.878) |  |
| Adjuvant chemotherapy |  |  | 0.034 |  | 0.025 |
| No | 226 (25.39%) | 46.0 |  | Reference |  |
|  |  |  |  |  |  |
| Yes | 664 (74.61%) | NA |  | 0.771 (0.614-0.968) |  |
| Data are presented as mean ±standard deviation or number (%).  CI, confidence interval; Co-PaL, the combined prealbumin and lymphocyte; DFS, disease free survival; HR, hazard ratio; MV, multivariate analysis; NA, not available; NLR, neutrophil-to-lymphocyte ration; PNI, prognostic nutritional index; UV, univariate analysis.  * Tumor stages are based on 8th edition of the Union for International Cancer Control TNM classification.  † Defined as Clavien-Dindo grade II or greater. | | | | | |
