# Supplementary material for: Validation of the predictive value of combined prealbumin and lymphocyte score for prognosis of stage II/III gastric cancer following curative resection
Source: Front Oncol. 2026 Feb 12;16:1650351. doi: 10.3389/fonc.2026.1650351 (PMC12935669; doi:10.3389/fonc.2026.1650351)
Supplement: Supplementary file 3 [file Table2.doc]

| **Supplementary Table 2.** Clinicopathological characteristics of the entire cohort (n = 1621) | | | |
| --- | --- | --- | --- |
| Variables | Training group  (n = 731) | Validation group  (n = 890) | *P* value |
| Gender (males) | 492 (67.31%) | 565 (63.48%) | 0.108 |
| Age (years) | 54.55 ±10.96 | 57.30 ±11.18 | <0.001 |
| Body Mass Index (kg/m^2^) | 21.48 ±2.91 | 22.18 ±3.21 | <0.001 |
| Any comorbidities | 214 (29.27%) | 264 (29.66%) | 0.865 |
| Pre-operative lymphocyte count (×10^9^/L) | 1.76 ±0.67 | 1.74 ±0.64 | 0.443 |
| Pre-operative albumin level (g/L) | 36.86 ±4.68 | 40.51 ±4.46 | <0.001 |
| Pre-operative prealbumin level(mg/L) | 197.05 ±69.22 | 230.90 ±67.91 | <0.001 |
| Pre-operative hemoglobin (g/L) | 116.07 ±24.85 | 118.84 ±25.99 | 0.030 |
| PNI score | 45.68 ±6.21 | 49.21 ±6.10 | <0.001 |
| Co-PaL score |  |  | <0.001 |
| 0 | 290 (39.67%) | 453 (50.90%) |  |
| 1 | 306 (41.86%) | 347 (38.99%) |  |
| 2 | 135 (18.47%) | 90 (10.11%) |  |
| Neo-adjuvant chemotherapy | 0 | 128 (14.38%) | <0.001 |
| Operation method |  |  | <0.001 |
| Open | 691 (94.53%) | 498 (55.96%) |  |
| Laparoscopy | 40 (5.47%) | 392 (44.04%) |  |
| Type of resection |  |  | <0.001 |
| Distal subtotal gastrectomy | 503 (68.81%) | 603 (67.75%) |  |
| Proximal subtotal gastrectomy | 35 (4.79%) | 9 (1.01%) |  |
| Total gastrectomy | 193 (26.40%) | 278 (31.24%) |  |
| Harvested lymph node number | 19.55 ±8.36 | 20.54 ±7.51 | 0.012 |
| pTNM stage* |  |  | 0.020 |
| II | 190 (25.99%) | 278 (31.24%) |  |
| III | 541 (74.01%) | 612 (68.76%) |  |
| Intra-operative blood loss (mL) | 208.28 ±142.98 | 189.09 ± 133.36 | 0.005 |
| Operation time (min) | 205.82 ±56.22 | 187.25 ±58.18 | <0.001 |
| Peri-operative blood transfusion | 170 (23.26%) | 179 (20.11%) | 0.125 |
| Post-operative complications † |  |  | 0.027 |
| None | 660 (90.29%) | 772 (86.74%) |  |
| Yes | 71 (9.71%) | 118 (13.26%) |  |
| Post-operative hospital stays (days) | 13.96 ±4.94 | 11.55 ±6.06 | <0.001 |
| Adjuvant chemotherapy (yes) | 545 (74.56%) | 664 (74.61%) | 0.981 |
| Data are presented as mean ±standard deviation or number (%).  Co-PaL, the combined prealbumin and lymphocyte; PNI, prognostic nutritional index.  ^†^ Defined as Clavien-Dindo grade II or greater.  ^*^ Tumor stages are based on 8th edition of the Union for International Cancer Control TNM classification. | | | |
